# The gut-eye axis: the retinal/ocular degenerative diseases and the emergent therapeutic strategies

**DOI:** 10.3389/fncel.2024.1468187

**Published:** 2024-09-26

**Authors:** Sonda Kammoun, Mona Rekik, Aryj Dlensi, Samir Aloulou, Walid Smaoui, Sahla Sellami, Khaled Trigui, Rahma Gargouri, Imen Chaari, Hayet Sellami, Dhawia Elatoui, Nahed Khemakhem, Ines Hadrich, Sourour Neji, Balkiss Abdelmoula, Nouha Bouayed Abdelmoula

**Affiliations:** ^1^Genomics of Signalopathies at the Service of Precision Medicine LR23ES07 FMS, University of Sfax, Sfax, Tunisia; ^2^Ophthalmology Department, Faculty of Medicine, Habib Bourguiba University Hospital, University of Sfax, Sfax, Tunisia; ^3^Medical Carcinology Department, Faculty of Medicine, Mohamed Ben Sassi University Hospital of Gabes, University of Sfax, Sfax, Tunisia; ^4^Urology Department, Faculty of Medicine, Habib Bourguiba University Hospital, University of Sfax, Sfax, Tunisia; ^5^Drosophila Research Unit UR22ES03 FMS, University of Sfax, Sfax, Tunisia; ^6^Parasitology and Mycology Department, Faculty of Medicine, University of Sfax, Sfax, Tunisia; ^7^Fungal and Parasitic Molecular Biology Laboratory LR05ES11 FMS, University of Sfax, Sfax, Tunisia

**Keywords:** gut-eye axis, gut microbiota, microbiota, degenerative eye diseases, retinopathies

## Abstract

The interplay between human microbiota and various physiological systems has garnered significant attention in recent years. The gut microbiota plays a critical role in maintaining physiological homeostasis and influences various aspects of human health, particularly via the gut brain axis. Since 2017, the challenging concept of the gut-retina axis has emerged thanks to a network analysis emphasizing the potential role of the gut microbiota disruption in the development of the age-related macular degeneration and further retinal damages. Many other ocular disorders have been linked to the dysbiosis of the gut microbiota, including uveitis and glaucoma. It has been shown that age related macular degeneration can be prevented or reversed using a diet that induces changes in the gut microbiota. The potential link between the gut microbiota as well as others types of microbiota such as the ocular surface microbiota and the development/progression of age related as well as inherited retinal degenerations and other degenerative eye diseases, has recently been broadened. Therefore, the pathogenesis of several eye diseases has recently been associated with a larger perception called the gut eye axis. This mini-review examines the potential mechanisms underlying the gut eye axis and suggests implications for the management of eye diseases. By understanding the modulation of the gut microbiota and its impact on eye disease, this mini-review provides insight into potential therapeutic interventions and avenues for future research.

## 1 Introduction

Through dynamic bidirectional communication along the gut-brain axis, the gut microbiota interacts with the host to regulate the development and optimal function of all body systems, (digestive and extra-digestive) (Chaudhry et al., 2023). This communication network involves a complex interplay of neural, hormonal and immune signaling mechanisms, connecting the gut and the brain (Zheng et al., 2023). Imbalance between gut microbial communities, or dysbiosis, and alterations in the processes of this network of signaling pathways via inflammatory, metabolic and oxidative stress mechanisms, etc., have been implicated in a number of human health disorders. Recent advances in microbiota research have unveiled intricate connections between the gut microbiota composition and the pathogenesis of systemic diseases, including metabolic disorders, autoimmune conditions, and neurological disorders including developmental and degenerative conditions of the nervous system (Intili et al., 2023; Ullah et al., 2023). In parallel, studies have begun to unravel the potential implications of gut microbiota dysbiosis in the context of eye health and disease. In fact, the microbiota-retina axis emerged as a concept when a network analysis was conducted to highlight the likely role of gut microbiota disruption in the development of retinal damages (Rowan et al., 2017; Rinninella et al., 2018). Rowan’s study found that age-related macular degeneration (AMD) can be prevented or reversed by using a diet that induces changes in the gut microbiota. The potential link between the gut microbiota as well as the ocular surface microbiota and ocular health has recently been broadened (Scuderi et al., 2022; Spörri et al., 2024). Therefore, the pathogenesis of several eye diseases, including AMD, diabetic retinopathy (DR), glaucoma, uveitis and newly inherited retinal degenerations (Douglas et al., 2023), etc.… has recently been associated with a larger concept termed the gut-eye axis (Floyd and Grant, 2020).

This mini-review of the literature aims to provide an overview of the current understanding of the gut-eye axis and the potential mechanisms underlying this axis, to offer insights into the biological basis of this relationship. It aims to disclose the role of the gut microbiota in the development, progression and management of retinopathies as well as other degenerative eye diseases including glaucoma and uveitis; and finally to elucidate challenges to target the gut microbiota to improve the prevention and treatment of these eye diseases.

## 2 Gut microbiota

The gut microbiota is involved in digestion, vitamin production, and the synthesis of short-chain fatty acids (SCFAs) like acetate, propionate, and butyrate. It also protects against harmful bacteria, supports immune system development, and helps regulate T-cell populations like regulatory T cells (Tregs), T helper 1 (Th1) cells, and Th17 cells. These effects are mediated by the microbiota and their byproducts, such as SCFAs from microbes like Bacteroides that regulate Tregs and Th17 cells (Kho and Lal, 2018; Zeng and Chi, 2015).

The gut microbiota comprises more than 100 trillion microorganisms associated with multiple functions, from nutrient metabolism to protection against pathogens. Each individual encompasses a unique gut microbiota profile that changes over time, depending on certain variables such as lifestyle, physical exercise, body mass index, and dietary habits. Advances in sequencing technology, have played a key role in determining the composition of microbiota, which has been found to be mainly dominated by Proteobacteria, followed by Actinobacteria, Firmicutes, and Bacteroidetes (Xue et al., 2021).

A healthy gut microbiota maintains a balance between beneficial and harmful bacteria, and imbalances are linked to autoimmune intestinal diseases like Crohn’s disease and ulcerative colitis (Hornef and Pabst, 2016; Viladomiu et al., 2017). Disruptions in the gut microbiota are associated with various conditions, including irritable bowel syndrome, inflammatory bowel disease, obesity, diabetes, multiple sclerosis, rheumatoid arthritis, graft-versus-host disease, and neurodegenerative diseases, some of which can have serious effects on eye health (Rajilić-Stojanović et al., 2015; Sheehan et al., 2015; Aron-Wisnewsky and Clément, 2016).

## 3 Gut-eye axis

Since the introduction of the gut-retina axis concept by Rowan et al., the importance of gut microbes as regulators of ocular disease has gained significant attention (Rowan et al., 2017; Jayasudha et al., 2020). Research has shown that diet, probiotics, and antibiotics can influence the gut microbiota and, in turn, affect the development of ocular diseases (Bringer et al., 2022). In addition, the gut microbiota has been increasingly recognized as a factor influencing the onset and progression of multiple ocular diseases, including uveitis, dry eye, macular degeneration, diabetic retinopathy and glaucoma (Fu et al., 2023). In this bibliometric study, Fu et al. found a total of 284 relevant publications from 2009 to 2023 about gut microbiota and eye diseases. There were few studies in the first 5 years, but the number has increased steadily since 2016. These studies involved 1,376 authors from 41 countries. The top journal and the most cited journal were both Investigative Ophthalmology & Visual Science. The author concluded that emerging findings suggest the existence of a gut-eye axis, wherein gut dysbiosis may be an important factor influencing the onset and progression of multiple ocular diseases (Fu et al., 2023). The gut microbiota has a metabolic activity similar to that exhibited by other organs of the human body (Xue et al., 2021) and produces various metabolites, which can affect the host’s physiology and pathology. These metabolites can enter the systemic circulation and reach the eye, where they can modulate the function of the eye and contribute to the development of ocular diseases (Napolitano et al., 2021; Zhang et al., 2023).

A recent study analyzed publications from the Web of Science Core Collection database and identified articles related to gut microbiota and ocular diseases, with an increasing trend in the number of publications over time (Moon et al., 2020a). The most studied and described microbiota dysbiosis is in the bacterial microbiota, but other agents, such as viral and fungal communities, also reside in the intestine and can lead to various eye diseases when deregulated (Moon et al., 2020a,b). The gut-eye axis hypothesis suggests that dysbiosis of the intestinal microbiota or disruption in the intestinal barrier may result in translocation of intestinal microbes and further impact the eye, which is remotely located from the gut (Moon et al., 2020c; Deng et al., 2021). The gut microbiota and their metabolites may serve as endogenous culprits of ocular diseases, exerting their function through molecular mimicry and integrated immunological pathways (Deng et al., 2021).

The specific mechanisms through which gut microbiota, considered as a modulator of systemic health, including ocular health, influence pathogenic processes in ocular diseases are the following:

- Systemic inflammation and immune response:
- Microbiota-derived metabolites: Gut microbiota produce various metabolites, such as SCFAs, which have systemic anti-inflammatory effects. SCFAs like butyrate can influence the systemic immune response by modulating T cell differentiation and function. Dysbiosis, or microbial imbalance, can lead to reduced production of beneficial SCFAs and increased systemic inflammation, which may contribute to chronic inflammatory ocular diseases like uveitis or AMD.
- Endotoxemia: The gut barrier dysfunction often associated with dysbiosis can lead to the translocation of bacterial endotoxins, such as lipopolysaccharides, into the bloodstream, a condition known as endotoxemia. These endotoxins can trigger systemic inflammation and exacerbate ocular inflammation and degeneration.
- Immune system modulation:
- Gut-Associated Lymphoid Tissue (GALT): The gut-associated lymphoid tissue plays a pivotal role in the development and regulation of systemic immunity. Microbial interactions within the GALT can shape the systemic immune response and influence the activation of immune cells that migrate to ocular tissues. For instance, altered microbiota composition may lead to an imbalance in regulatory T cells and pro-inflammatory Th17 cells, affecting ocular immune responses and potentially contributing to autoimmune ocular conditions.
- Cytokine release: Microbiota can modulate the production of cytokines and other immune mediators that influence inflammation. For example, microbiota-derived signals can impact the levels of cytokines such as interleukin-6 (IL-6) and tumor necrosis factor-alpha (TNF-alpha), which are involved in the inflammatory processes of ocular diseases like diabetic retinopathy.
- Blood-retina barrier integrity:
- Microbial metabolites and barrier function: Microbiota-derived metabolites, such as SCFAs, also affect the integrity of epithelial barriers throughout the body, including the blood-retina barrier. Disruption of this barrier can lead to retinal vascular leakage and contribute to conditions such as diabetic macular edema. The health of the gut barrier and its interaction with systemic and ocular barriers is a critical aspect of maintaining ocular health.
- Microbiota and oxidative stress:
- Oxidative stress modulation: The gut microbiota influences oxidative stress levels through the production of various metabolites and the modulation of systemic inflammation. Oxidative stress is a known contributor to retinal diseases such as AMD and retinal degenerative conditions. Dysbiosis can lead to increased oxidative stress and subsequent damage to retinal cells.
- Genetic and epigenetic interactions:
- Host-microbiota interactions: The interactions between host genetics and microbiota can also play a role in ocular diseases. Genetic variations in host receptors that interact with microbiota-derived molecules may influence susceptibility to ocular diseases. Additionally, microbiota can impact epigenetic modifications, potentially affecting gene expression related to inflammation and oxidative stress in ocular tissues.

## 4 Gut microbiota and ocular health/disease

Our literature review has identified a growing body of evidence supporting the influence of the gut microbiota on ocular health and disease. Research has demonstrated variations in the gut microbiota composition among individuals with certain eye diseases compared to healthy controls. Furthermore, preclinical studies have highlighted potential mechanisms by which the gut microbiota may regulate ocular immune responses, inflammation, and neuro-degeneration, thereby affecting the pathogenesis of eye diseases. However, the precise mechanisms of the gut-eye axis remain incompletely understood, necessitating further investigation.

### 4.1 Age-related macular degeneration

A research study indicated that individuals with advanced AMD showed signs of intestinal dysbiosis, characterized by a distinct composition of gut bacteria compared to healthy older adults. Specifically, the bacterial genera Anaerotruncus and Oscillibacter, as well as the species *Ruminococcus torques* and *Eubacterium ventriosum*, were found to be differentially abundant (Zinkernagel et al., 2017). Conversely, AMD subjects exhibited reduced levels of Oscillospira, Blautia, and Dorea compared to their healthy counterparts (Lin, 2018, 2019). Using high-throughput RNA sequencing in germ-free mice, Skondra and colleagues identified significant aspects of retinal gene regulation in AMD influenced by the gut microbiota (Nadeem et al., 2020). While the precise mechanisms underlying AMD pathogenesis remain poorly understood, inflammatory pathways linked to innate immunity have been implicated. Two prevailing hypotheses suggest that microbial imbalances may contribute to AMD progression through immune dysregulation and altered nutrient absorption.

In AMD pathogenesis, regulated immune activation involving the recruitment of microglia and macrophages to the sub retinal and choroidal regions, mast cell activation, and the retinal pigment epithelium (RPE) immune responses are thought to be significant (Handa et al., 2019). The gut microbiota could potentially modulate inflammation and microglial function during AMD development and progression.

### 4.2 Diabetic retinopathy

DR, a vision-threatening complication of diabetes, can lead to blindness if not well-managed (Cheung et al., 2010). Recent studies have shown a close association between gut microbiota dysbiosis and DR. Firstly, gut microbiota has been found to play a role in retinal neurodegeneration (Dong et al., 2021; Beli et al., 2018), inflammatory processes (Yang et al., 2021), glucose metabolism, insulin resistance, and entero-insulin secretion (Iatcu et al., 2021). On the other hand, alterations in the gut bacterial microbiota in individuals with DR can also contribute to the onset and progression of the condition (Das et al., 2021). For instance, carnosine, an endogenous dipeptide with significant antioxidant and anti-inflammatory properties, is depleted in DR patients compared to healthy controls (Caruso et al., 2019). These findings suggest that gut microbiota mediates gut-retinal communication.

Modifications in the gut microbiota can trigger pathological inflammation and exacerbate DR progression, impacting both the local immune system within the gut and the overall systemic immune response (Tilg et al., 2020). Specifically, heightened intestinal permeability and the resulting microbial translocation have been identified as key factors in the onset and development of DR (Rizzetto et al., 2018).

### 4.3 Uveitis

Research using animal models has revealed substantial variations in intestinal bacteria composition in cases of uveitis, implying that gut modifications may establish specific conditions that could potentially be modified through the use of probiotics (Xue et al., 2021).

In clinical settings, imbalances in gut bacterial populations have been observed in individuals with uveitis (Kalyana Chakravarthy et al., 2018). Furthermore, research has shown that transferring the gut microbiota from patients with Behcet’s disease and Vogt-Koyanagi-Harada disease, both of which involve multi organ inflammation including uveitis, significantly worsens disease severity in recipient experimental autoimmune uveitis mice (Ye et al., 2018, 2020).

### 4.4 Glaucoma

Given the role of CD4+ T cells and the microbiota in the development of glaucoma, there is a recent proposal to classify glaucoma within the autoimmune disease spectrum (Geyer and Levo, 2020). Chen et al. (2018) conducted a remarkable study indicating that a temporary elevation in intraocular pressure can trigger auto reactive T cells to enter the retina, with these T cells being primed by the symbiotic microbiota. Recent research has highlighted the significance of commensal microbiota dysbiosis, encompassing disruptions in gut and oral microbiota, in the initiation of neurological disorders and progressive neuronal degeneration (Chaudhry et al., 2023).

Several studies have demonstrated a potential link between gut microbiota and glaucoma. Notably, individuals with primary open-angle glaucoma exhibit distinct gut microbiota compositions and serum metabolites compared to healthy individuals (Gong et al., 2020). Butyrate, a metabolite produced by gut bacteria, has been found to reduce intraocular pressure in rats independently of changes in blood pressure (Skrzypecki et al., 2018). Furthermore, an elevated level of trimethylamine, a uremic toxin generated by gut microbiota, has been detected in the aqueous humor of glaucoma patients (Skrzypecki et al., 2021).

During glaucoma, the microbiota of the eye (ocular surface and intra-ocular cavity) and the digestive system (from the mouth to the intestine via the estomace) are different from those of healthy patients. However, a huge gap remains in our understanding of the mechanisms linking microbiota and glaucoma as a neurodegenerative disease (Hernández-Zulueta et al., 2024; Wu et al., 2024).

## 5 The role of gut microbiota in preventing ocular diseases

Targeting the gut microbiota to prevent and manage eye diseases represents a promising frontier in medical research, expanding our understanding of the intricate relationship between gut health and ocular function.

As we have already seen, recent investigations have highlighted how dysbiosis, or imbalance in gut microbial communities, can contribute to the development and progression of eye conditions such as AMD, DR, glaucoma, and uveitis. In fact, the gut microbiota, comprising trillions of microorganisms, plays an important role beyond digestive functions, influencing systemic inflammation, immune modulation, and even neurologic processes.

The therapeutic potential of targeting the gut microbiota lies in its ability to modulate these inflammatory and metabolic pathways. Interventions such as dietary modifications, probiotics, fecal microbiota transplantation, or microbial-derived therapies hold promise in restoring microbial balance, reducing systemic inflammation, and improving outcomes in ocular diseases. By understanding and manipulating these microbial communities, clinicians may potentially personalize treatments to mitigate disease progression and enhance therapeutic efficacy (Parker et al., 2022).

Moreover, ongoing research efforts are exploring the identification of microbial biomarkers predictive of ocular disease risk or progression, which could revolutionize early diagnosis and intervention strategies (Wang et al., 2023; Khan et al., 2021). In a recent study, genetic analyses have determined that gut microbiota is causally associated with myopia risk. Furthermore, transplantation of fecal microbiota from healthy donors into a mouse model of myopia results in increased plasma 3-IAA levels and a simultaneous delay in myopia progression, as well as improved maintenance of type I alpha 1 collagen (COL1A1) expression in the sclera. The authors suggest new strategies for intervening in high myopia by remodeling the intestinal microbiota and supplementing with indole (Li et al., 2024). This multidisciplinary approach not only integrates microbiology and ophthalmology but also underscores the importance of systemic health in maintaining optimal eye function. As our understanding of the gut-eye axis continues to evolve, so too does the potential for innovative therapies that harness the power of the gut microbiota to promote ocular health.

## 6 Critical discussion

While this review provides a comprehensive summary of the existing literature on the gut-eye axis and its implications for retinal and ocular degenerative diseases, a more robust critical analysis is necessary to enhance its impact.

- Study methodologies: Many studies rely on animal models that may not fully replicate human conditions. Variability in experimental protocols and differences in model systems can lead to inconsistent results. Future research should aim to standardize methodologies and incorporate more human-relevant models to improve the translational potential of findings.
- Mechanistic understanding: While correlations between gut health and retinal degeneration are increasingly recognized, the underlying mechanisms remain poorly understood. There is a need for more detailed mechanistic studies to elucidate how gut-derived factors influence ocular health at a molecular level.
- Longitudinal data: Much of the current research is cross-sectional, providing a snapshot rather than a detailed picture of how gut-eye interactions evolve over time. Longitudinal studies are essential to understand the progression of ocular diseases in relation to gut health.
- Clinical relevance: Many findings from preclinical studies have yet to be validated in clinical settings. There is a critical need for clinical trials to assess the efficacy and safety of potential therapeutic interventions targeting the gut-eye axis.
- Heterogeneity in patient populations: Studies often overlook the heterogeneity of patient populations, including variations in genetics, diet, and other environmental factors that might influence the gut-eye relationship. Future research should consider these variables to develop personalized therapeutic strategies.

## 7 Directions for future research

To address these limitations and advance the field, future research should focus on the following directions:

- Enhanced model systems: Develop and use advanced models and organ-on-a-chip technologies to better mimic human physiology and disease conditions.
- Mechanistic investigations: Conduct in-depth studies to uncover the specific pathways through which gut microbiota and gut-derived metabolites impact retinal health.
- Longitudinal and multicenter studies: Implement longitudinal studies and multicenter trials to track disease progression and validate findings across diverse populations.
- Clinical translation: Prioritize the translation of preclinical findings into clinical practice by designing and executing well-structured clinical trials.
- Personalized approaches: Explore personalized medicine approaches by integrating genetic, environmental, and lifestyle factors into research and treatment strategies.

By addressing these critical areas, future research can contribute to a more nuanced understanding of the gut-eye axis and facilitate the development of targeted, effective therapeutic interventions for retinal and ocular degenerative diseases.

## 8 Conclusion

These findings of the literature review underscore the complex interplay between gut microbiota and ocular health, suggesting potential avenues for therapeutic interventions and disease management.

Modulation of gut microbiota represents a promising approach for targeting systemic inflammation and immune dysregulation associated with certain eye diseases. Additionally, the identification of microbial biomarkers predictive of ocular disease risk or progression may facilitate early diagnosis and personalized treatment strategies.

Nonetheless, several challenges and unanswered questions persist, warranting continued research efforts to unravel the intricacies of the gut-eye axis and its therapeutic implications.

## References

[ref1] Aron-Wisnewsky J. Clément K. (2016). The gut microbiome, diet, and links to cardiometabolic and chronic disorders. Nat. Rev. Nephrol. 12, 169–181. doi: 10.1038/nrneph.2015.191 26616538

[ref2] Beli E. Yan Y. Moldovan L. Vieira C. P. Gao R. Duan Y.. (2018). Restructuring of the gut microbiome by intermittent fasting prevents retinopathy and prolongs survival in db/db mice. Diabetes 67, 1867–1879. doi: 10.2337/db18-0158, PMID: 29712667 PMC6110320

[ref3] Bringer M. A. Gabrielle P. H. Bron A. M. Creuzot-Garcher C. Acar N. (2022). The gut microbiota in retinal diseases. Exp. Eye Res. 214:108867. doi: 10.1016/j.exer.2021.108867 34856206

[ref4] Caruso G. Fresta C. G. Fidilio A. O'Donnell F. Musso N. Lazzarino G.. (2019). Carnosine decreases PMA-induced oxidative stress and inflammation in murine macrophages. Antioxidants (Basel, Switzerland) 8:281. doi: 10.3390/antiox8080281, PMID: 31390749 PMC6720685

[ref5] Chaudhry T. S. Senapati S. G. Gadam S. Mannam H. P. S. S. Voruganti H. V. Abbasi Z.. (2023). The impact of microbiota on the gut-brain axis: examining the complex interplay and implications. J. Clin. Med. 12:5231. doi: 10.3390/jcm12165231, PMID: 37629273 PMC10455396

[ref6] Chen H. Cho K. S. Vu T. H. K. Shen C. H. Kaur M. Chen G.. (2018). Commensal microflora-induced T cell responses mediate progressive neurodegeneration in glaucoma. Nat. Commun. 9:3209. doi: 10.1038/s41467-018-05681-9, PMID: 30097565 PMC6086830

[ref7] Cheung N. Mitchell P. Wong T. Y. (2010). Diabetic retinopathy. Lancet (London, England) 376, 124–136. doi: 10.1016/S0140-6736(09)62124-3 20580421

[ref8] Das T. Jayasudha R. Chakravarthy S. Prashanthi G. S. Bhargava A. Tyagi M.. (2021). Alterations in the gut bacterial microbiome in people with type 2 diabetes mellitus and diabetic retinopathy. Sci. Rep. 11:2738. doi: 10.1038/s41598-021-82538-0, PMID: 33531650 PMC7854632

[ref9] Deng Y. Ge X. Li Y. Zou B. Wen X. Chen W.. (2021). Identification of an intraocular microbiota. Cell Discovery 7:13. doi: 10.1038/s41421-021-00245-6, PMID: 33750767 PMC7943566

[ref10] Dong L. Zhang Z. Liu X. Wang Q. Hong Y. Li X.. (2021). RNA sequencing reveals BMP4 as a basis for the dual-target treatment of diabetic retinopathy. J. Mol. Med. (Berl) 99, 225–240. doi: 10.1007/s00109-020-01995-8, PMID: 33188599

[ref11] Douglas V. P. Douglas K. A. A. Iannaccone A. (2023). Microbiome and inherited retinal degenerations. Am. J. Pathol. 193, 1669–1674. doi: 10.1016/j.ajpath.2023.03.005, PMID: 37024045

[ref12] Floyd J. L. Grant M. B. (2020). The gut-eye axis: lessons learned from murine models. Ophthalmol Therapy 9, 499–513. doi: 10.1007/s40123-020-00278-2 PMC7406577 32617914

[ref13] Fu X. Tan H. Huang L. Chen W. Ren X. Chen D. (2023). Gut microbiota and eye diseases: a bibliometric study and visualization analysis. Front. Cell. Infect. Microbiol. 13:1225859. doi: 10.3389/fcimb.2023.1225859, PMID: 37621873 PMC10445766

[ref14] Geyer O. Levo Y. (2020). Glaucoma is an autoimmune disease. Autoimmun. Rev. 19:102535. doi: 10.1016/j.autrev.2020.102535 32234407

[ref15] Gong H. Zhang S. Li Q. Zuo C. Gao X. Zheng B.. (2020). Gut microbiota compositional profile and serum metabolic phenotype in patients with primary open-angle glaucoma. Exp. Eye Res. 191:107921. doi: 10.1016/j.exer.2020.107921, PMID: 31917963

[ref16] Handa J. T. Bowes Rickman C. Dick A. D. Gorin M. B. Miller J. W. Toth C. A.. (2019). A systems biology approach towards understanding and treating non-neovascular age-related macular degeneration. Nat. Commun. 10:3347. doi: 10.1038/s41467-019-11262-1, PMID: 31350409 PMC6659646

[ref17] Hernández-Zulueta J. Bolaños-Chang A. J. Santa Cruz-Pavlovich F. J. Valero Rodríguez A. D. Lizárraga Madrigal A. del Rio-Murillo X.. (2024). Microbial dynamics in ophthalmic health: exploring the interplay between human microbiota and Glaucoma pathogenesis. Medicina (Kaunas) 60:592. doi: 10.3390/medicina60040592, PMID: 38674238 PMC11051970

[ref18] Hornef M. W. Pabst O. (2016). Real friends: *Faecalibacterium prausnitzii* supports mucosal immune homeostasis. Gut 65, 365–367. doi: 10.1136/gutjnl-2015-310027, PMID: 26531718

[ref19] Iatcu C. O. Steen A. Covasa M. (2021). Gut microbiota and complications of Type-2 diabetes. Nutrients 14:166. doi: 10.3390/nu14010166, PMID: 35011044 PMC8747253

[ref20] Intili G. Paladino L. Rappa F. Alberti G. Plicato A. Calabrò F.. (2023). From dysbiosis to neurodegenerative diseases through different communication pathways: an overview. Biology 12:195. doi: 10.3390/biology12020195, PMID: 36829474 PMC9952972

[ref21] Jayasudha R. Das T. Kalyana Chakravarthy S. Sai Prashanthi G. Bhargava A. Tyagi M.. (2020). Gut mycobiomes are altered in people with type 2 diabetes mellitus and diabetic retinopathy. PLoS One 15:e0243077. doi: 10.1371/journal.pone.0243077, PMID: 33259537 PMC7707496

[ref22] Kalyana Chakravarthy S. Jayasudha R. Sai Prashanthi G. Ali M. H. Sharma S. Tyagi M.. (2018). Dysbiosis in the gut bacterial microbiome of patients with uveitis, an inflammatory disease of the eye. Indian J. Microbiol. 58, 457–469. doi: 10.1007/s12088-018-0746-9, PMID: 30262956 PMC6141402

[ref23] Khan R. Sharma A. Ravikumar R. Parekh A. Srinivasan R. George R. J.. (2021). Association between gut microbial abundance and sight-threatening diabetic retinopathy. Invest. Ophthalmol. Vis. Sci. 62:19. doi: 10.1167/iovs.62.7.19, PMID: 34132747 PMC8212427

[ref24] Kho Z. Y. Lal S. K. (2018). The human gut microbiome – a potential controller of wellness and disease. Front. Microbiol. 9:1835. doi: 10.3389/fmicb.2018.01835, PMID: 30154767 PMC6102370

[ref25] Li H. Du Y. Cheng K. Chen Y. Wei L. Pei Y.. (2024). Gut microbiota-derived indole-3-acetic acid suppresses high myopia progression by promoting type I collagen synthesis. Cell Discovery 10:89. doi: 10.1038/s41421-024-00709-5 39187483 PMC11347609

[ref26] Lin P. (2018). The role of the intestinal microbiome in ocular inflammatory disease. Curr. Opin. Ophthalmol. 29, 261–266. doi: 10.1097/ICU.0000000000000465, PMID: 29538183

[ref27] Lin P. (2019). Importance of the intestinal microbiota in ocular inflammatory diseases: a review. Clin. Exp. Ophthalmol. 47, 418–422. doi: 10.1111/ceo.13493, PMID: 30834680

[ref28] Moon J. Choi S. H. Yoon C. H. Kim M. K. (2020b). Gut dysbiosis is prevailing in Sjögren's syndrome and is related to dry eye severity. PLoS One 15:e0229029. doi: 10.1371/journal.pone.0229029, PMID: 32059038 PMC7021297

[ref29] Moon J. Ryu J. S. Kim J. Y. Im S. H. Kim M. K. (2020c). Effect of IRT5 probiotics on dry eye in the experimental dry eye mouse model. PLoS One 15:e0243176. doi: 10.1371/journal.pone.0243176, PMID: 33259525 PMC7707591

[ref30] Moon J. Yoon C. H. Choi S. H. Kim M. K. (2020a). Can gut microbiota affect dry eye syndrome? Int. J. Mol. Sci. 21:8443. doi: 10.3390/ijms21228443 33182758 PMC7697210

[ref31] Nadeem U. Xie B. Movahedan A. D’Souza M. Barba H. Deng N.. (2020). High throughput RNA sequencing of mice retina reveals metabolic pathways involved in the gut-retina axis. bioRxiv 2020. doi: 10.1101/2020.10.01.318949

[ref32] Napolitano P. Filippelli M. Davinelli S. Bartollino S. dell'Omo R. Costagliola C. (2021). Influence of gut microbiota on eye diseases: an overview. Ann. Med. 53, 750–761. doi: 10.1080/07853890.2021.1925150, PMID: 34042554 PMC8168766

[ref33] Parker A. Romano S. Ansorge R. Aboelnour A. Le Gall G. Savva G. M.. (2022). Fecal microbiota transfer between young and aged mice reverses hallmarks of the aging gut, eye, and brain. Microbiome 10:68. doi: 10.1186/s40168-022-01243-w, PMID: 35501923 PMC9063061

[ref34] Rajilić-Stojanović M. Jonkers D. M. Salonen A. Hanevik K. Raes J. Jalanka J.. (2015). Intestinal microbiota and diet in IBS: causes, consequences, or epiphenomena? Am. J. Gastroenterol. 110, 278–287. doi: 10.1038/ajg.2014.427, PMID: 25623659 PMC4317767

[ref35] Rinninella E. Mele M. C. Merendino N. Cintoni M. Anselmi G. Caporossi A.. (2018). The role of diet, micronutrients and the gut microbiota in age-related macular degeneration: new perspectives from the gut-retina axis. Nutrients 10:1677. doi: 10.3390/nu10111677, PMID: 30400586 PMC6267253

[ref36] Rizzetto L. Fava F. Tuohy K. M. Selmi C. (2018). Connecting the immune system, systemic chronic inflammation and the gut microbiome: the role of sex. J. Autoimmun. 92, 12–34. doi: 10.1016/j.jaut.2018.05.008, PMID: 29861127

[ref37] Rowan S. Jiang S. Korem T. Szymanski J. Chang M. L. Szelog J.. (2017). Involvement of a gut-retina axis in protection against dietary glycemia-induced age-related macular degeneration. Proc. Natl. Acad. Sci. USA 114, E4472–E4481. doi: 10.1073/pnas.1702302114 28507131 PMC5465926

[ref38] Scuderi G. Troiani E. Minnella A. M. (2022). Gut microbiome in retina health: the crucial role of the gut-retina Axis. Front. Microbiol. 12:726792. doi: 10.3389/fmicb.2021.726792, PMID: 35095780 PMC8795667

[ref39] Sheehan D. Moran C. Shanahan F. (2015). The microbiota in inflammatory bowel disease. J. Gastroenterol. 50, 495–507. doi: 10.1007/s00535-015-1064-1 25808229

[ref40] Skrzypecki J. Izdebska J. Kamińska A. Badowska J. Przybek-Skrzypecka J. Bombuy J.. (2021). Glaucoma patients have an increased level of trimethylamine, a toxic product of gut bacteria, in the aqueous humor: a pilot study. Int. Ophthalmol. 41, 341–347. doi: 10.1007/s10792-020-01587-y 32914277 PMC7840632

[ref41] Skrzypecki J. Żera T. Ufnal M. (2018). Butyrate, a gut bacterial metabolite, lowers intraocular pressure in normotensive but not in hypertensive rats. J. Glaucoma 27, 823–827. doi: 10.1097/IJG.0000000000001025 30001267

[ref42] Spörri L. Uldry A. C. Kreuzer M. Herzog E. L. Zinkernagel M. S. Unterlauft J. D.. (2024). Exploring the ocular surface microbiome and tear proteome in glaucoma. Int. J. Mol. Sci. 25:6257. doi: 10.3390/ijms25116257, PMID: 38892444 PMC11172891

[ref43] Tilg H. Zmora N. Adolph T. E. Elinav E. (2020). The intestinal microbiota fuelling metabolic inflammation. Nat. Rev. Immunol. 20, 40–54. doi: 10.1038/s41577-019-0198-4, PMID: 31388093

[ref44] Ullah H. Arbab S. Tian Y. Liu C. Q. Chen Y. Qijie L.. (2023). The gut microbiota-brain axis in neurological disorder. Front. Neurosci. 17:1225875. doi: 10.3389/fnins.2023.1225875, PMID: 37600019 PMC10436500

[ref45] Viladomiu M. Kivolowitz C. Abdulhamid A. Dogan B. Victorio D. Castellanos J. G.. (2017). IgA-coated *E. coli* enriched in Crohn's disease spondyloarthritis promote TH17-dependent inflammation. Sci. Transl. Med. 9:eaaf9655. doi: 10.1126/scitranslmed.aaf9655 28179509 PMC6159892

[ref46] Wang Q. Wu S. Ye X. Tan S. Huang F. Su G.. (2023). Gut microbial signatures and their functions in Behcet's uveitis and Vogt-Koyanagi-Harada disease. J. Autoimmun. 137:103055. doi: 10.1016/j.jaut.2023.103055, PMID: 37208257

[ref47] Wu Y. Shi R. Chen H. Zhang Z. Bao S. Qu J.. (2024). Effect of the gut microbiome in glaucoma risk from the causal perspective. BMJ Open Ophthalmol. 9:e001547. doi: 10.1136/bmjophth-2023-001547, PMID: 38286567 PMC10826588

[ref48] Xue W. Li J. J. Zou Y. Zou B. Wei L. (2021). Microbiota and ocular diseases. Front. Cell. Infect. Microbiol. 11:759333. doi: 10.3389/fcimb.2021.759333, PMID: 34746029 PMC8566696

[ref49] Yang G. Wei J. Liu P. Zhang Q. Tian Y. Hou G.. (2021). Role of the gut microbiota in type 2 diabetes and related diseases. Metab. Clin. Exp. 117:154712. doi: 10.1016/j.metabol.2021.154712 33497712

[ref50] Ye Z. Wu C. Zhang N. Du L. Cao Q. Huang X.. (2020). Altered gut microbiome composition in patients with Vogt-Koyanagi-Harada disease. Gut Microbes 11, 539–555. doi: 10.1080/19490976.2019.1700754, PMID: 31928124 PMC7524263

[ref51] Ye Z. Zhang N. Wu C. Zhang X. Wang Q. Huang X.. (2018). A metagenomic study of the gut microbiome in Behcet's disease. Microbiome 6:135. doi: 10.1186/s40168-018-0520-6, PMID: 30077182 PMC6091101

[ref52] Zeng H. Chi H. (2015). Metabolic control of regulatory T cell development and function. Trends Immunol. 36, 3–12. doi: 10.1016/j.it.2014.08.003, PMID: 25248463 PMC4280284

[ref53] Zhang J. Y. Greenwald M. J. Rodriguez S. H. (2023). Gut microbiome and retinopathy of prematurity. Am. J. Pathol. 193, 1683–1690. doi: 10.1016/j.ajpath.2023.01.013 36780985

[ref54] Zheng Y. Bonfili L. Wei T. Eleuteri A. M. (2023). Understanding the gut-brain axis and its therapeutic implications for neurodegenerative disorders. Nutrients 15:4631. doi: 10.3390/nu15214631, PMID: 37960284 PMC10648099

[ref55] Zinkernagel M. S. Zysset-Burri D. C. Keller I. Berger L. E. Leichtle A. B. Largiadèr C. R.. (2017). Association of the intestinal microbiome with the development of neovascular age-related macular degeneration. Sci. Rep. 7:40826. doi: 10.1038/srep40826, PMID: 28094305 PMC5240106

